# Climate Change and Prevention – Review of Prevention Indicators of the German Federal States in Relation to ‘Climate Change and Health’

**DOI:** 10.25646/13411

**Published:** 2025-09-17

**Authors:** Veronika Reisig, Anne Starker, Marjolein Haftenberger, Marie-Hélène Manz, Klaus Möhlendick, Kristin Mühlenbruch, Annkathrin Haar, Angelina Taylor, Brigitte Borrmann

**Affiliations:** 1 Bavarian Health and Food Safety Authority, Department of Health Reporting, Social Medicine, Public Health Service, Oberschleißheim, Germany; 2 Robert Koch Institute, Department 2: Epidemiology and Health Monitoring, Berlin, Germany; 3 Senate Department for Science, Health, Care and Equality, Department Health, Berlin, Germany; 4 Ministry of Labour, Social Affairs, Women and Health of Saarland, Unit E1, Saarbrücken, Germany; 5 Thuringian Ministry of Social Affairs, Health, Labour and Family, Division for Health Promotion, Addiction Services, Pact for Public Health Service, Erfurt, Germany; 6 State Office for Occupational Safety, Consumer Protection and Health Brandenburg, Division of Health, Potsdam, Germany; 7 State Office for Health and Occupational Safety North Rhine-Westphalia, Health Reporting Section, Bochum, Germany

**Keywords:** Climate change, Health, Prevention, Health reporting, Prevention reporting, Indicators, Indicator systems, Checklist, German federal states, Germany, Public health

## Abstract

**Background:**

The risks of climate change for human health are becoming increasingly apparent. The prevention indicator system of the German federal states (Länder in Deutschland), developed between 2018 and 2022, was therefore reviewed in relation to its relevance to climate change.

**Methods:**

As a first step, a working group with members from different German federal states developed a model on the relationships between climate change and health in the context of prevention. Central aspects of this model were translated into a checklist based on guiding questions, which was used to conduct a systematic, standardised, and evidence-informed assessment of the climate relevance of the prevention indicator system of the German federal states.

**Results:**

Climate change relevance was identified for a total of 49 out of 73 prevention indicators. Most frequently, climate relevance was found for indicators relating to particularly vulnerable groups to climate change-related health impacts (27 indicators), followed by 18 indicators addressing health consequences of climate change.

**Conclusions:**

The assessment methodology that we developed proved suitable and can be applied to assess climate relevance in other health indicator systems. This prevention indicator system requires further development of climate aspects that have not yet been included, such as ‘health-relevant climate change impacts’, ‘health costs’, and indicators on vaccine-preventable diseases as climate adaptation measures.

## 1. Introduction

The increasing impacts of climate change, such as higher average temperatures and more frequent extreme weather events like heatwaves, floods, or droughts, promote the emergence and spread of diseases within the population. At the same time, they increase the health risks for individuals affected by pre-existing health burdens [1–4].

Climate change affects human health and wellbeing both directly and indirectly. Direct effects include heat-related illnesses or injuries resulting from extreme weather events [1]. Indirect health impacts arise, for example, from the increased spread of disease-carrying vectors such as certain mosquitoes or ticks, elevated air pollution and pollen levels [5, 6], potential impairments in water supply and quality [7], threats to food security due to crop failures [1, 8], as well as challenges related to climate migration and habitat loss [9, 10]. Fundamentally, climate change affects all people and entails the aforementioned risks to public health. However, these risks are unevenly distributed across the population depending on numerous factors such as age, pre-existing conditions, working conditions, place of residence, and social status [11]. Particular vulnerability arises when multiple risks accumulate. In light of the increasing disease burden and growing threats to human health, it is becoming more important for the health sector and health policy to engage with the links between climate change and health.

The 95th Conference of Health Ministers (Gesundheitsministerkonferenz, GMK) on June 22–23, 2022, tasked the working groups of the Association of the Highest State Health Authorities (Arbeitsgemeinschaft der Obersten Landesgesundheitsbehörden, AOLG) with addressing the topic of ‘Climate Change and Health’ [12]. The Prevention Indicators Subgroup (Unterarbeitsgruppe Präventionsindikatoren, UAG) of the AOLG Working Group on Health Reporting, Prevention, Rehabilitation, and Social Medicine (Arbeitsgruppe Gesundheitsberichterstattung, Prävention, Rehabilitation und Sozialmedizin, AG GPRS) also carried out this mandate (Infobox 1).

**Key messages**

► A model was developed that illustrates the relationships between climate change and health in the context of prevention, which can also be used to assess climate relevance in other health indicator systems.
► Based on this, the climate relevance of the prevention indicator system of the German federal states was determined in a standardised and transparent manner using a systematic ex-post assessment procedure with guiding questions and a checklist.
► Approximately two-thirds of the 73 indicators in the prevention indicator system of the German federal states have a climate relevance.
► Most frequently, climate relevance was found for indicators relating to particularly vulnerable population groups (27 indicators).
► 18 indicators address health consequences of climate change, nine indicators focus on climate mitigation measures, eight indicators cover causes of climate change, and three indicators concern climate adaptation measures.

**Infobox 1**

The diagram illustrates the structure of the Conference of Health Ministers (Gesundheitsministerkonferenz, GMK), a specialised ministerial conference attended by the health ministers or health senators of the German federal states (Länder in Deutschland), along with the subordinate working bodies [13].

**Figure:**
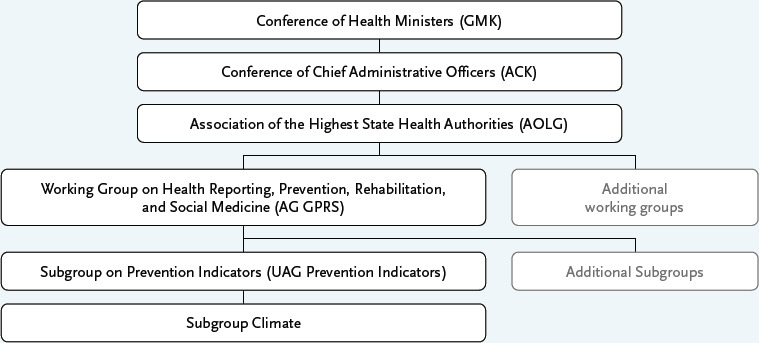

**Infobox 2 Thematic Areas of the Prevention Indicator System of the German Federal States**

The 73 indicators of the prevention indicator system are distributed across 14 thematic areas, encompassing prevention-relevant living conditions and behaviours, risks and resources, as well as morbidity and mortality.

► Thematic area 1 – Context factors (6 indicators)
► Thematic area 2 – Setting-related factors (5 indicators)
► Thematic area 3 – Psychosocial resources (7 indicators)
► Thematic area 4 – Health literacy (1 indicator)
► Thematic area 5 – Physical activity (11 indicators)
► Thematic area 6 – Nutrition (9 indicators)
► Thematic area 7 – Tobacco and alcohol (9 indicators)
► Thematic area 8 – Vaccination (3 indicators)
► Thematic area 9 – Overweight and obesity (2 indicators)
► Thematic area 10 – Diabetes (2 indicators)
► Thematic area 11 – Cardiovascular diseases (4 indicators)
► Thematic area 12 – Cancer (4 indicators)
► Thematic area 13 – Mental illnesses (8 indicators)
► Thematic area 14 – Life expectancy and premature mortality (2 indicators)

A detailed overview of the prevention indicator system can be found in the final report of the UAG Prevention Indicators [14] and in Annex Table 1.

The Subgroup on Prevention Indicators developed a prevention indicator system consisting of 73 indicators between 2018 and 2022 to enable coordinated and comparable prevention reporting across the German federal states (Infobox 2) [13]. The selection of indicators prioritised public health relevance and amenability to intervention through health promotion and prevention measures; health-related aspects of climate change were initially not specifically considered. Of the 73 indicators in the prevention indicator system, 46 focus on population-based monitoring of the prevalence of health risks and preventable diseases. The remaining 27 indicators include structural or contextual indicators as well as mortality-related outcome indicators. However, work on the prevention indicator system is not yet complete; for some indicators, operationalisation is still ongoing and/or necessary data sources are not yet available. The indicator system was applied in the federal states’ contributions to the 2023 Prevention Report of the National Prevention Conference. Using type 2 diabetes as an example, a causal chain was illustrated in order to demonstrate possible prevention approaches, starting with health-relevant contextual factors, setting-related factors, resource and behavioural factors, and ending with health outcomes relating to diabetes [13].

To implement the aforementioned resolution of the GMK and in response to the growing relevance of climate-related aspects concerning population health, the AG GPRS tasked the UAG Prevention Indicators with reviewing whether the prevention indicators previously developed by the UAG are relevant to climate change and, if so, to identify the specific relationships (Work Package 1). Building on this, the indicator system was to be supplemented with several key prevention indicators that are meaningful in the context of climate change (Work Package 2). This paper addresses the first work package, the ex-post assessment of the existing 73 indicators of the prevention indicator system regarding their links to climate change. The aim was to explore whether and how the already existing indicators of the prevention indicator system can be understood in relation to climate change, health, prevention, and health promotion. This must be differentiated from the narrower question of whether indicators of the prevention indicator system validly represent specific climate change aspects – this question was not the focus of the work. To our knowledge, this represents the first assessment of an existing health-related indicator system for its climate change relevance. It complements national and international initiatives and efforts to strengthen monitoring and surveillance of climate-related health aspects. At the international level, for example, the Lancet Countdown reports on the impacts of ongoing climate change on health and political responses using over 50 indicators [15]. In Europe, the indicators of the European Climate and Health Observatory are notable [16], an initiative of the European Environment Agency in cooperation with the European Commission, particularly the Directorate-General for Climate Action (DG CLIMA). Furthermore, individual countries such as Austria and Australia have also developed extensive monitoring systems in recent years [17, 18]. In Germany, the project Monitoring of Climate Change-related Health Aspects (MOCCHA) was recently initiated to develop a nationwide indicator set for monitoring climate-relevant health aspects [19]. Additionally, the German Adaptation Strategy to Climate Change (Deutsche Anpassungsstrategie an den Klimawandel, DAS) foresees the identification of nationwide indicators for the field of action ‘human health and care’ [20]. Many of the above-mentioned initiatives applied assessment criteria for indicator selection to ensure that these are, for example, scientifically sound, relevant, based on valid data sources, reproducible, reliable, and feasible [21]. Comparable criteria were also applied in the development of the prevention indicator system. However, a new procedure was required for the ex-post assessment of this system regarding potential climate change relevance.

## 2. Methods

### 2.1 Conceptual preparations

**Figure 1:**
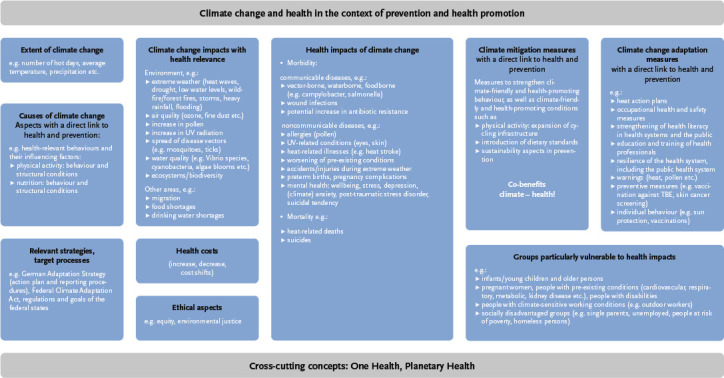
Model of ‘Climate Change and Health in the Context of Prevention and Health Promotion’

To address the two work packages mentioned above, the Climate Subgroup (KG Klima) was established in June 2023 under the UAG Prevention Indicators (Infobox 1). This subgroup consists of members of the UAG Prevention Indicators and the Climate Change and Health Office at the Robert Koch Institute (RKI). Between summer 2023 and spring 2025, the subgroup focused on assessing the prevention indicator system for its relevance to climate change. Initially, a conceptual framework was developed to enable a systematic and standardised approach to examining the 73 existing prevention indicators for their climate relevance. As a first step, a review of conceptualisations concerning the links between climate change and health was conducted. This aimed to break down the term ‘climate change’ into individual subcomponents and schematically position them within the field of prevention and health promotion. Building on exemplary models [1, 2, 22], a simple conceptual model was first created, including the components ‘Causes of climate change’, ‘Characteristics/extent of climate change’, ‘Climate change impacts relevant to health’, ‘Health consequences of climate change’, and ‘Health costs’. To embed the model within the action-oriented and value-based context of health promotion and prevention, it was expanded to include the component ‘Particularly vulnerable groups with regard to health impacts’, as these represent important target groups for preventive measures. Additionally, the sub-areas ‘Ethical aspects’, ‘Climate mitigation measures’, ‘Climate adaptation measures’, and ‘Relevant strategies and target processes’ were incorporated. Based on the literature [1, 2, 5–7, 11, 22–33] all components were illustrated with example details (Figure 1).

### 2.2 Development of the Assessment Methodology

The model described above served as the starting point for developing a tool to assess the climate relevance of the prevention indicators. The aim was to enable an approach that was systematic, standardised, and pragmatic in equal measure. Through a consensus process within the Climate Subgroup (KG Klima), key aspects of the developed model (Figure 1) were translated into a series of guiding questions, which were used to determine the climate relevance of the existing prevention indicators, taking into account the evidence base (Table 1). Particular attention was given to identifying indicators that describe co-benefits, i.e., aspects that can positively influence both climate and health, such as physical activity or dietary behaviours [32, 34, 35]. Climate relevance of an indicator was deemed present if there was a connection to at least one of the areas covered by the guiding questions, with the practical relevance of the link also being considered. If a theoretically conceivable connection existed between the indicator and any of the assessed areas, but which was not proven or supported in the literature, then no climate relevance was attributed for that area. Relevant sources used as the evidence base for determining climate relevance included the Robert Koch Institute’s status report on climate change and health [1, 5–7, 23–32], the climate impact and risk analysis by the German Environment Agency (Umweltbundesamt) [11], indicators and reporting related to the German Adaptation Strategy on Climate Change (Deutsche Anpassungsstrategie an den Klimawandel) [36], and the 2022 report of the Intergovernmental Panel on Climate Change (IPCC) [37]. The assessment outcome was recorded dichotomously as either ‘climate relevance present’ or ‘climate relevance not present’. There was consensus within the Climate Subgroup that a more nuanced grading of climate relevance would create an illusion of precision that could not be justified within the pragmatically designed assessment process and was unnecessary for the intended purpose of the investigation. Moreover, the assessment did not aim to provide a comparative ranking regarding the strength of the relationship. For example, identifying an indicator as related to a cause of climate change does not imply a judgement on how significant that cause is compared to others, some of which may not be represented in the prevention indicator system. The final step in developing the assessment method involved compiling the individual guiding questions into a checklist. This checklist served as the central tool for conducting the assessment, documenting the results, and recording the underlying evidence base.

### 2.3 Procedure for assessing climate relevance

The 73 prevention indicators available were examined for their climate relevance using the checklist, and the assessments were agreed upon within the Subgroup Climate (KG Klima). This also included eleven so-called developmental indicators, for which operationalisation had not yet been finalised and/or suitable data sources could not be identified. During the first application of the checklist, several aspects were refined, such as the specification of the wording of the guiding questions, the evidence base to be applied, and clarification of certain terminologies. In particular, within the model component ‘Groups particularly vulnerable to climate-related health impacts’, the concept of vulnerability was defined in greater detail. Vulnerability was understood as resulting from one or more of the following aspects: exposure (e.g. to heat), susceptibility (e.g. due to pre-existing illness), and adaptive capacity (e.g. limited as a result of socioeconomic disadvantage) [30]. After reviewing all indicators using the revised checklist, the assessment outcomes were considered as a whole to ensure consistency in the application of the guiding questions and internal coherence of the ratings. Where necessary, assessments were refined in an iterative process.

## 3. Results

Overall, a climate relevance could be identified for around two-thirds of the indicators in the prevention indicator system. These indicators are presented and explained below according to their allocation to the components of the model ‘Climate change and health in the context of prevention and health promotion’ (Figure 1). The 49 indicators with a climate relevance could be assigned to the components ‘Particularly vulnerable groups’, ‘Health impacts of climate change’, ‘Climate adaptation measures’, ‘Climate mitigation measures’, ‘Causes of climate change’ and ‘Ethical aspects’. Some indicators were assigned to two components. They are therefore presented in two different subsections below, with the respective justifications provided. No separate presentation is made for the six indicators assigned to the category ‘Ethical aspects’, as this allocation was consistently made in addition to the category ‘Particularly vulnerable groups due to socioeconomic factors’ and was uniformly justified by reference to health equity and/or climate justice considerations.

No indicators from the prevention indicator system were assigned to the model components ‘Extent of climate change’, ‘Climate change impacts relevant to health’, ‘Health costs’ and ‘Relevant strategies/policy processes’.

The prevention indicator system was not originally developed as an indicator framework specifically focused on climate and health. The indicators allocated to the individual model components below therefore do not cover the full spectrum of indicators that would be relevant for each component, but represent only those included within the prevention indicator system.

### 3.1 Climate relevance: Model component ‘Causes of climate change’

A link to the model component ‘Causes of climate change’ – and thus the potential for co-benefits – was identified for the following eight indicators (Table 2).

### 3.2 Climate relevance: Model component ‘Health impacts of climate change’

For 18 of the prevention indicators, a climate relevance in terms of ‘Health impacts of climate change’ was identified (Table 3).

### 3.3 Climate relevance: Model component ‘Climate mitigation measures’

This category includes nine indicators that describe health-promoting measures which also contribute to climate mitigation. Four of these indicators relate to nutritional standards in communal catering across different settings (Table 4).

### 3.4 Climate relevance: Model component ‘Climate adaptation measures’

Climate adaptation measures can be integrated into setting-based health promotion, and certain vaccinations may become more important in the context of climate change. Against this background, three indicators were identified as having climate relevance in terms of ‘Climate adaptation measures’ (Table 5).

### 3.5 Climate relevance: Model component ‘Particularly vulnerable groups’

Most of the indicators with climate relevance – a total of 27 – were assigned to the component ‘Particularly vulnerable groups’. These indicators describe groups that are especially at risk of adverse health impacts of climate change due to socioeconomic or health-related factors. The following table is therefore subdivided accordingly (Table 6, Part A and Part B). The indicators in Table 6, Part A can also be assigned to the component ‘Ethical aspects’ (health equity, environmental justice).

## 4. Discussion

At the federal state level, the Prevention Indicators Subgroup, which is part of the AOLG Working Group on Health Reporting and Prevention (AG GPRS), examined the extent to which the indicators contained in the federal states’ prevention indicator system are linked to climate change. To develop an assessment procedure, a model was first created, drawing on current national and international analyses of the interrelations between climate change and health. From this model, guiding questions were derived and transferred into a checklist in order to enable allocation to aspects of climate change and to systematically assess the climate relevance of the indicators.

As a result of the assessment, 49 of the 73 prevention indicators were found to have a climate relevance: 27 indicators capture population groups whose health is particularly at risk from the impacts of climate change and were therefore assigned to the model component ‘Particularly vulnerable groups’. Eighteen indicators were linked to the component ‘Health impacts of climate change’, nine to ‘Climate mitigation measures’, eight to ‘Causes of climate change’, and three to ‘Climate adaptation measures’. The eight indicators related to the causes of climate change are of particular importance, as measures that positively influence these causes can bring benefits both for climate mitigation and for population health. Although climate change was not explicitly taken into account in the development of the indicator system, around two-thirds of all indicators in the prevention indicator system show a climate relevance. This underlines the need to strengthen consideration of climate change as a cross-cutting issue in relation to population health.

### 4.1 Challenges in the assessment

Answering the guiding questions for evaluating the climate relevance of the prevention indicators did not always produce a clear result, as the climate relevance of an indicator may relate to several components of the underlying model (Figure 1). Moreover, the relationships may be bidirectional and are highly dependent on the precise definition and operationalisation of each indicator.

#### Multiple allocations

For certain indicators, climate relevance could be identified for more than one component of the underlying model. An example is the indicator ‘12-month prevalence of coronary heart disease’, which can be assigned both to the model component ‘Health impacts of climate change’ (increase in heart attacks and deaths from heart attacks, partly due to heat extremes, rising air pollution and chronic stress) and to the component ‘Particularly vulnerable groups’ (increased risk of climate-related health impacts among people with pre-existing coronary heart disease, for example due to thermoregulatory stress leading to increased cardiac strain, blood pressure drop and even circulatory collapse). Such multiple allocations also occurred with indicators of other disease prevalence, such as mental disorders. Other examples include the indicators ‘State programmes for health promotion in nurseries’ and ‘State programmes for health promotion in schools’, which could be assigned both to ‘Climate mitigation measures’ and ‘Climate adaptation measures’. In total, 22 indicators were allocated to more than one category.

#### Bidirectional allocations

The assessment of climate relevance also showed that indicators may capture both positive and negative impacts. This is illustrated by the indicator ‘Degree of urbanisation’: a high degree of urbanisation can be associated with extensive land sealing and the related negative effects on the local climate. At the same time, however, a high level of urbanisation through densification within cities may also help prevent urban sprawl into surrounding areas, which – especially if combined with climate-friendly solutions for infill development – can have positive effects on climate-related factors. Based on the definition of this indicator, which does not cover qualitative aspects of urbanisation or knock-on effects in surrounding areas, a clear determination of the direction of its climate relevance is not possible, but also not required for the purposes of this analysis.

#### Importance of the definition and operationalisation of indicators for their climate assessment

During the assessment process it became clear that the exact definition and operationalisation of an indicator – that is, the measure applied – are crucial for the evaluation outcome. This can be seen, for example, in the indicator ‘Fruit and vegetable consumption’. The indicator is defined as the ‘proportion of the population with daily fruit and/or vegetable intake’ and is based on survey data on the frequency of fruit and vegetable consumption. The indicator is associated with healthy dietary habits and was therefore included in the prevention indicator set. At the same time, it could also be linked to a diet lower in meat and more climate-friendly. However, based on the reported frequency of fruit and vegetable intake alone, it cannot be inferred that people are necessarily consuming a low-meat diet, nor that their fruit and vegetable consumption is climate-friendly – such as by preferring local, seasonal, organically grown, or unpackaged products. Studies highlight the complex links between production methods and climate impact [82–84], which means that the climate relevance may present itself in different ways. Thus, the differentiated consideration of the indicator fruit and vegetable consumption illustrates that a sound assessment of its climate relevance would require a more precise capture of the climate-related aspects of diet.

A special status is accorded to the development indicators of the prevention indicator system (Annex Table 1), where the indicator definitions and/or operationalisation have not yet been fully developed. These primarily include indicators that describe living conditions and structures, such as ‘walkability’ or ‘cycling friendliness’. Despite the absence of precise definitions or operationalisation in some cases, these indicators were included in the evaluation process and assessed based on their apparent intent. However, this assessment should be considered provisional, pending the further specification of the indicators.

### 4.2 Strengths and Limitations

The checklist has proven to be a suitable tool for assessing climate relevance. Based on this framework, all 73 indicators of the German federal states’ prevention indicator system could be evaluated. The systematic, standardised, and evidence-informed approach has proven practical, and an additional valuable application of the prevention indicator system could be identified.

Nevertheless, regarding the methodology of the evaluation process, it should be noted that no systematic literature review could be conducted on the diverse climate relevance aspects for the 73 indicators under review. Associated biases cannot be ruled out. Decisions on climate relevance were made based on the available literature through a multi-step consensus process among members of the Climate Subgroup. Given the complexity of the topic of climate change and health, it quickly became evident that scaling the assessment results would unnecessarily complicate the process without contributing to understanding. However, even the dichotomous evaluation of climate relevance only reflects the literature that was reviewed and should therefore be regarded as a snapshot. New scientific findings related to climate change and climate adaptation may alter these assessments in the future.

It is also important to emphasise that the federal states’ prevention indicators were not developed with the primary aim of capturing climate change and health, but rather according to other criteria, such as general public health relevance and the amenability to intervention [14]. The evaluation results therefore represent a valuable added benefit of the prevention indicator system and can serve as a starting point for comprehensive reporting on climate change and climate adaptation that takes prevention into account. Furthermore, the evaluation provides a tool that can be used to assess climate relevance when adapting the prevention indicator system or, if applicable, other indicator systems. Consequently, a comparison with other national and international indicator systems – particularly those developed specifically to capture climate change or its impacts on human health – would be worthwhile.

### 4.3 Conclusion and Outlook

The Climate Subgroup of the UAG Prevention Indicators has completed the review of the existing prevention indicator system with regard to its climate relevance (Work Package 1). An integrative approach of this kind, which systematically relates the analysis of the health aspects of climate change to a prevention indicator system, has not previously been pursued. The comprehensive evaluation of the indicators and the associated discussions have shown that the definitions of the existing prevention indicators are not always clearly designed to assess potential climate relevance, and specific gaps in the prevention indicator system have become apparent. While various components of the model are represented, certain areas – for example, vaccine-preventable infectious diseases linked to climate change – are currently insufficiently covered. Furthermore, although mental health is represented by numerous indicators, existing indicators do not specifically address mental health burdens associated with climate change or climate adaptation. No indicators could be identified within the model components ‘Extent/Aspects of Climate Change’, ‘Climate Change Impacts with Health Relevance’, or ‘Health Costs’ in the prevention indicator system. This highlights a further need for specific indicators that both demonstrate prevention potential and clearly reflect climate relevance, particularly for indicators that exhibit co-benefits. In a next step, new prevention indicators are to be developed to cover relevant climate change-related health aspects and to address identified gaps in the prevention indicator system with respect to the model components on climate change (Work Package 2). Ideas for this have already been collected during the completed evaluation process. Additionally, existing indicators from other national and international indicator systems, such as those from the Lancet Countdown, are to be considered [15], and the work of individual countries, such as Austria and Australia, will also be taken into account. Furthermore, ongoing processes for the development of indicators and indicator sets in Germany that are of particular relevance are being incorporated, with an emphasis on achieving synergies and consistency, including the aforementioned health indicators for Germany’s Climate Adaptation Strategy [20] and the German project Monitoring of Climate Change-related Health Aspects (MOCCHA) [19].

## Figures and Tables

**Table 1:** Guiding questions for assessing the climate relevance of prevention indicators and specific examples for interpreting the guiding questions in line with the checklist

Does the indicator describe …	
… the causes of climate change?	e.g. climate-friendly or climate-damaging physical activity or dietary behaviours, or their determinants (structural conditions); potential co-benefits for health and climate may be identified here
… the extent or specific aspects of climate change?	e.g. number of heat days, average temperature, precipitation
… the climate change impacts relevant to health?	e.g. extreme weather events such as heatwaves or storms, air quality issues such as ozone, particulate matter, or pollen exposure, ultraviolet (UV) radiation, spread of disease vectors
... potential health consequences of climate change?	e.g. communicable diseases (such as vector-borne, waterborne, or foodborne diseases), noncommunicable diseases (such as allergic, UV-related or heat-related conditions, accidents/injuries during extreme weather events, mental health disorders), or (e.g. heat-related) mortality
… health costs resulting from climate change?	e.g. costs associated with the aforementioned diseases
... climate mitigation measures directly related to health, prevention, or health promotion (co-benefits)?	e.g. measures to strengthen climate- and health-promoting conditions (such as expanding cycling infrastructure, implementing climate-friendly nutritional standards, integrating sustainability in prevention measures) and individual behavioural adaptations (such as active transport and more plant-based diets)
... climate adaptation measures directly related to health, prevention, or health promotion?	e.g. heat action plans, occupational health and safety, strengthening climate-related health literacy, education and training, resilience within the (public) health system, early warning services for heat or pollen, preventive measures such as vaccination against tick-borne encephalitis (TBE), skin cancer screening, or sun protection
… groups particularly vulnerable to climate-related health impacts?	e.g. infants, young children, older people, pregnant women, individuals with pre-existing conditions/with disabilities/with climate-sensitive working conditions, vulnerable and marginalised groups (e.g. single parents, and people who are unemployed, at risk of poverty, or who are homeless)
... other aspects in the context of climate and health?	e.g. links to overarching or cross-cutting aspects such as ethical dimensions (e.g. health equity, environmental justice), relevant strategies or policy processes, overarching concepts (One Health, Planetary Health)

**Table 2:** Indicators with climate relevance ‘Causes of climate change’

Indicator	Climate relevance
**Degree of urbanisation** Degree of urbanisation according to Eurostat in three groups: cities, towns and suburbs, rural areas	Urbanisation may be associated with land sealing, air pollutants, and energy and resource consumption. At the same time, depending on infrastructure measures, it can also contribute to efficient resource use with regard to buildings, water and energy [38, 39].
**Recreational space** Recreational space per inhabitant in m^2^	Recreational areas (including sports grounds, parks and campsites) and cemeteries are largely green, less sealed surfaces. They can absorb and store carbon dioxide from the atmosphere. Vegetation in recreational areas helps regulate temperature by providing shade and lowering ambient temperature through evaporative cooling. In addition, they improve air quality by absorbing pollutants and producing oxygen [40].
**Walkability** Walkability index	Car-oriented urban infrastructure significantly contributes to climate change. Measures to improve walkability and cycling friendliness contribute both to climate mitigation and to the promotion of physical activity and health [41–43].
**Cycling friendliness/cycling infrastructure** Index of cycling friendliness/cycling infrastructure	
**Transport-related physical activity** Proportion of the population that meets or exceeds physical activity recommendations through active travel	
**Mode share** Proportion of trips and passenger kilometres by mode of transport	
**Breastfeeding** Breastfeeding prevalence and average duration	Formula feeding requires energy and resources for production, transport and preparation, and generates packaging waste. By contrast, breastfeeding is a climate-friendly and sustainable form of infant nutrition. Low breastfeeding rates contribute to climate change [44–46].
**Tobacco consumption** Proportion of the population that currently uses tobacco daily	High levels of tobacco consumption require increased tobacco production, which affects agricultural land use, deforestation, and subsequently greenhouse gas emissions. Another factor with high levels of tobacco use is the energy-intensive production of packaging materials [47].

**Table 3:** Indicators with climate relevance ‘Health impacts of climate change’

Indicator	Climate relevance
**Life expectancy and healthy life expectancy** Average life expectancy at birth and in good health	Extreme weather events, increased impacts of air pollution, and the spread of climate-related diseases may reduce life expectancy [48]. Healthy life expectancy may also decline as climate-related health problems such as respiratory diseases, cardiovascular diseases, and mental disorders increase [1]. In particularly vulnerable groups, such as people with pre-existing conditions [27, 30], premature mortality is expected to rise [27, 30].
**Premature mortality** Years of life lost before the age of 65	
**Breast cancer mortality in women** Number of deaths from breast cancer per 100,000 women	Increased mortality among seriously ill patients at regional level can be triggered by extreme weather events that severely disrupt regional infrastructure or health services, leading for example to delayed operations and interrupted chemotherapy. People with cancer are among the groups most dependent on a functioning healthcare infrastructure [49, 50].
**Self-rated health-related quality of life** Proportion of the population with high self-rated health-related quality of life	Self-rated quality of life, psychological distress, and chronic stress are influenced by changing climatic conditions. In particular, the increasing frequency and intensity of extreme weather events such as heatwaves, floods and storms place considerable strain on psychosocial wellbeing. Studies show that chronic stress reactions, up to and including clinically relevant mental disorders, are increasing as a result of climate change [7, 29].
**Self-rated psychological distress** Proportion of the population reporting high psychological distress	
**Self-rated stress/chronic stress** Proportion of the population reporting high stress levels	
**Mental health problems in children and adolescents** Proportion of children and adolescents with mental health problems	Mental disorders can arise or be worsened as a result of climate change. Studies show a significant increase in post-traumatic and/or depressive symptoms, anxiety symptoms, suicide attempts, and substance misuse, particularly following extreme weather events [29, 33]. Evidence also indicates links between air pollution and mental disorders such as depression, attention deficit hyperactivity disorder (ADHD), and schizophrenia [29, 51, 52]. Children and adolescents are especially vulnerable to the psychological consequences [53]. Moreover, long-term uncertainties associated with climate change, such as economic losses or migration, intensify chronic stress reactions including sleep disturbances [29, 54]. Consequently, incapacity for work, rehabilitation, and early retirement due to mental disorders are likely to increase as their prevalence rises with climate change.
**Sleep disorders** Proportion of the population with difficulties falling asleep or staying asleep	
**Depressive symptoms** Proportion of the population with depressive symptoms	
**Outpatient diagnoses of mental disorders** Proportion of statutory health insurance members with outpatient diagnoses of dementia, depression, and phobic or other anxiety disorders	
**Work incapacity due to mental disorders** Average number of work incapacity days due to mental disorders per 1,000 employed persons	
**Rehabilitation due to mental disorders** Proportion of pension insurance members undergoing rehabilitation for mental disorders	
**Early retirement due to mental disorders** Proportion of pension insurance members taking early retirement due to mental disorders	
**Suicides** Number of suicides per 100,000 inhabitants	
**12-month prevalence of hypertension** Proportion of the population with physician-diagnosed hypertension	Cardiovascular diseases are influenced by climate change. Studies show increasing morbidity and mortality – particularly for heart attacks and strokes. This rise is attributable both to direct effects, such as heat exposure and air pollution, and to indirect mechanisms, such as chronic stress reactions [7, 55, 56].
**12-month prevalence of coronary heart disease** Proportion of the population with physician-diagnosed coronary heart disease	
**12-month prevalence of stroke** Proportion of the population with physician-diagnosed stroke	
**Hospital morbidity: myocardial infarction, angina pectoris and stroke** Inpatient cases per 100,000 inhabitants	

**Table 4:** Indicators with climate relevance ‘Climate mitigation measures’

Indicator	Climate relevance
**Certified breastfeeding promotion in maternity clinics** Proportion of maternity clinics with certified breastfeeding promotion	Certified breastfeeding promotion can increase breastfeeding rates and duration. This, in turn, reduces the use of formula milk. The resulting avoidance of CO_2_ emissions from formula production, the reduction of packaging materials and plastic bottles, and the elimination of emissions from transport and storage can have a positive impact on the climate [46]. The Nurses Climate Challenge Europe (2022) supports this view and highlights the global climate relevance of breastfeeding promotion [57].
**Breastfeeding** Breastfeeding prevalence and average duration	
**Tobacco tax** Minimum tax rates, taxed quantities, and tax revenue for tobacco products	Higher tobacco taxation can reduce tobacco consumption, leading to a decrease in CO_2_ emissions associated with tobacco production and disposal [58].
**Implementation of nutritional standards in nurseries, schools, workplace canteens, and residential care for seniors** Proportion of nurseries, schools, companies, and care institutions for seniors that are committed to the quality standards of the German Nutrition Society (DGE)	Implementation of DGE standards in communal catering incorporates sustainability, seasonality, and regionality [59–62]. These standards promote a balanced, plant-based diet, which generally has a lower CO_2_ footprint than a diet high in meat [63]. As a result, they both promote health and protect the climate [64]. As climate mitigation measures, these programmes can also foster education and awareness of climate-friendly behaviours, for example by imparting knowledge on sustainable nutrition or environmentally friendly mobility [65].
**State programmes for health promotion in nurseries** Proportion of nurseries participating in the state programme relative to all nurseries in the region	State programmes for health promotion in nurseries and schools can foster education and awareness of climate-friendly behaviours, for example by imparting knowledge on sustainable nutrition or environmentally friendly mobility [65].
**State programmes for health promotion in schools** Proportion of schools participating in the state programme relative to all schools in the region	

**Table 5:** Indicators with climate relevance ‘Climate adaptation measures’

Indicator	Climate relevance
**State programmes for health promotion in nurseries** Proportion of nurseries participating in the state programme relative to all nurseries in the region	State-level health promotion programmes have considerable potential for integrating climate adaptation measures into nursery and school settings, and some of this is already evident in existing programmes [66–71]. These programmes can help strengthen the resilience of children and young people to climate-related health risks. This may be achieved through promoting health-conscious behaviour, strengthening the immune system via healthy nutrition and physical activity, and imparting knowledge about coping with heat or other extreme weather events.
**State programmes for health promotion in schools** Proportion of schools participating in the state programme relative to all schools in the region	
**Vaccination coverage at school entry examination for STIKO-recommended vaccinations** Proportion of children with initiated and completed primary immunisation for the vaccinations recommended by the Standing Committee on Vaccination (Ständige Impfkommission, STIKO).	Among the vaccinations recommended by STIKO for infants and children [72], there is a climate relevance for vaccination against rotaviruses and tetanus: waterborne infections such as rotavirus may increase following extreme weather events [24], and floods may also increase the risk of tetanus cases [73]. Monitoring coverage of these vaccinations is therefore useful as part of climate change adaptation measures.

**Table 6:** Indicators with climate relevance ‘Particularly vulnerable groups’

**A) Particularly vulnerable groups due to socioeconomic factors**	
**Indicator**	**Climate relevanc**
**Single-parent families** Proportion of single-parent households	Single parents, unemployed persons and persons with a low formal education belong to high-risk groups for poverty. People with limited socioeconomic and educational resources – and therefore reduced adaptive capacity – may be more exposed to pollutants and heat due to poor housing conditions. In single-parent and low-income families, infants and young children are particularly vulnerable to climate-related health impacts because of their heightened sensitivity to heat [30]. This can result in cumulative risks.
**Children and adolescents under 15 years in households receiving SGB II (Social Code II) benefits** Proportion per 100 inhabitants under 15 years	
**School leavers without a final qualification** Proportion of school leavers from general education schools without a basic school-leaving certificate	
**Unemployment rate** Proportion of unemployed individuals relative to the total labour force	
**Child poverty risk rate** Proportion of 0- to 6-year-olds living in households with an equivalised income below the at-risk-of-poverty threshold	
**Socioeconomic deprivation** Index of regional deprivation, including education, occupation and income	Climate change is expected to exacerbate social and health inequalities [74]. More deprived regions have fewer resources to implement adaptation measures and build resilient structures. Many people living in deprived regions are particularly vulnerable to the impacts of climate change. Existing disparities in cumulative health burdens may be amplified by structural deprivation [30].
**B) Climate relevance: Particularly vulnerable groups due to health-related factors**	
**Indicator**	**Climate relevance**
**Tobacco consumption** Proportion of the population that currently uses tobacco daily	Smoking increases the risk of respiratory and other chronic conditions, which may worsen during heatwaves or as a result of climate-related air pollution [27].
**Body Mass Index (BMI) in adults** Proportion of the adult population by WHO BMI categories	Obesity, due to a relatively low body surface-to-mass ratio, reduces heat dissipation because less fluid is emitted through sweating. It may also impair heat perception. In children, thermoregulation is not yet fully developed, making the negative effects of higher body weight more pronounced [27, 30, 65].
**Body Mass Index (BMI) in children** Proportion of children by BMI categories (Kromeyer-Hauschild reference) assessed at school entry examination	
**Incidence of diabetes** Proportion of adults with statutory health insurance newly diagnosed with diabetes within a year	Diabetes mellitus impairs skin blood circulation, reducing the effectiveness of sweating. This can lead to quicker overheating as well as blood sugar fluctuations with risk of hypo- or hyperglycaemia. Diabetes is also associated with impaired thermoregulatory lung responses. During heatwaves, the risk of hospitalisation increases [75].
**Prevalence of diabetes** Proportion of adults with statutory health insurance diagnosed with diabetes	
**12-month prevalence of hypertension** Proportion of the population with physician-diagnosed hypertension	Heat dilates blood vessels, lowering blood pressure. In combination with antihypertensive medication (e.g. diuretics), this may cause fainting (syncope), falls, or impaired organ perfusion leading to heart attacks [27].
**12-month prevalence of coronary heart disease** Proportion of the population with physician-diagnosed coronary heart disease	Extreme or prolonged heat causes thermoregulatory stress, leading to drops in blood pressure, increased cardiac strain, circulatory collapse or heatstroke [27]. Heatwaves raise the risk of heart attacks and strokes and are associated with higher mortality from these conditions [76, 77].
**12-month prevalence of stroke** Proportion of the population with physician-diagnosed stroke	
**Hospital morbidity: myocardial infarction, angina pectoris and stroke** Inpatient cases per 100,000 inhabitants	
**Incidence of colorectal cancer** Number of new colorectal cancer cases per 100,000 inhabitants per year	A cancer diagnosis has wide-ranging psychological and often socioeconomic consequences. As the disease progresses, many patients experience marked physical weakness. People with cancer are therefore particularly vulnerable to extreme heat [78]. Cancer-related immune suppression also heightens susceptibility to infections, including those linked to climate change.
**Incidence of cervical cancer** Number of new cervical cancer cases per 100,000 women per year	
**Incidence of lung cancer** Number of new lung cancer cases per 100,000 inhabitants per year	
**Nicotine-/tobacco-attributable diagnoses in hospital care** Inpatient cases per 100,000 inhabitants	Some tobacco-related conditions requiring hospital treatment are linked to increased vulnerability to heat and other climate-related impacts [27].
**Mental health problems in children and adolescents** Proportion of children and adolescents with mental health problems	Children are especially vulnerable to the psychological impacts of climate change, for instance during extreme weather events such as floods. Girls are a particular at-risk group, with evidence showing increased likelihood of anxiety disorders and substance misuse after experiencing natural disasters [53].
**Sleep disorders** Proportion of the population with difficulties falling asleep or staying asleep	Chronic stress burdens, including sleep disturbances, can heighten vulnerability to heat and other climate-related impacts [27, 33, 79–81]. Chronic psychological strain and mental disorders are also often associated with reduced capacity for individual adaptation. The risks are assumed to be even greater for long-term conditions requiring rehabilitation or leading to early retirement.
**Self-rated psychological distress** Proportion of the population reporting high psychological distress	
**Self-rated stress/chronic stress** Proportion of the population reporting high stress levels	
**Depressive Symptoms** Proportion of the population with depressive symptoms	
**Outpatient diagnoses of mental disorders** Proportion of statutory health insurance members with outpatient diagnoses of dementia, depression, and phobic or other anxiety disorders	
**Rehabilitation due to mental disorders** Proportion of pension insurance members undergoing rehabilitation for mental disorders	
**Early retirement due to mental disorders** Proportion of pension insurance members taking early retirement due to mental disorders	

## Appendix

**Annex Table 1:** Climate Relevance of the Indicators in the Prevention Indicator System of the German Federal States [14]

Indicator specification	Indicators	Climate relevance	Assignment
	**Thematic area 1 – Context factors**		
Core indicator^1^	Socio-economic deprivation	X	E and G
Core indicator	Single-parent families	X	E and G
Core indicator	Children and adolescents under 15 years in households receiving SGB II (Social Code II) benefits	X	E and G
Core indicator	School leavers without a final qualification	X	E and G
	Unemployment rate	X	E and G
	Child poverty risk rate	X	E and G
	**Thematic area 2 – Setting-related factors**		
	German Federal State programmes for health promotion in schools	X	C and D
	German Federal State programmes for health promotion in nurseries	X	C and D
Core indicator	Degree of urbanisation	X	A
Core indicator	Recreational spaced	X	A
	Workplace health management		
	**Thematic area 3 – Psychosocial** resources		
	Psychosocial resources of children and adolescents		
Core indicator	Social support		
	Self-efficacy expectation		
Core indicator	Self-rated health-related quality of life	X	B
	Self-rated mental stress	X	B and F
Core indicator	Self-rated stress/chronic stress	X	B and F
	Work-related stress experience		
	**Thematic area 4 – Health literacy**		
Core indicator	Health in the educational framework plan for nurseries/school		
	**Thematic area 5 – Physical activity**		
Development indicator^2^	Modal Split	X	A
Development indicator	Walkability	X	A
Development indicator	Cycling friendliness/cycling infrastructure	X	A
	Sports infrastructure		
Core indicator	School sports		
Development indicator	Physical activity as a component in the day-care curriculum		
Core indicator	Health-promoting physical activity		
Core indicator	Transport-related physical activity	X	A
	Sedentary behaviour		
	Work-related physical activity		
Core indicator	Motor coordination at school entry examination		
	**Thematic area 6 – Nutrition**		
Core indicator	Breastfeeding	X	A and C
	Certified breastfeeding promotion in maternity clinics	X	C
Core und Development indicator	Implementation of nutritional standards in nurseries	X	C
Development indicator	Implementation of nutritional standards in schools	X	C
Development indicator	Implementation of nutritional standards in workplace canteens	X	C
Development indicator	Implementation of nutrition standards in residential elderly care	X	C
Development indicator	Food prices		
Core indicator	Fruit and vegetable consumption		
Core indicator	Consumption of sugar-sweetened beverages/soft drinks		
	**Thematic area 7 – Tobacco and alcohol**		
	Tobacco tax	X	C
	Alcohol tax		
	Tobacco advertising		
Development indicator	Alcohol advertising		
Core indicator	Tobacco consumption	X	A and F
Core indicator	Alcohol consumption		
Core indicator	Nicotine-/tobacco-attributable diagnoses in hospital care	X	F
Core indicator	Alcohol-attributable diagnoses in hospital treatments		
	Alcohol-attributable diagnoses in deaths		
	**Thematic area 8 – Vaccination**		
Core indicator	Vaccination coverage at school entry examination for STIKO-recommended vaccinations	X	D
	Vaccination rate for complete HPV immunisation at age 15		
	Influenza vaccination in persons aged 60 and over		
	**Thematic area 9 – Overweight and obesity**		
Core indicator	Body Mass Index (BMI) in adults	X	F
Core indicator	Body Mass Index (BMI) in children	X	F
	**Thematic area 10 – Diabetes**		
	Diabetes incidence	X	F
Core indicator	Diabetes prevalence	X	F
	**Thematic area 11 – Cardiovascular diseases**		
Core indicator	12-month prevalence of hypertension	X	B and F
	12-month prevalence of coronary heart disease	X	B and F
	12-month prevalence of stroke	X	B and F
	Hospital morbidity: myocardial infarction, angina pectoris and stroke	X	B and F
	**Thematic area 12 – Cancer**		
	Breast cancer mortality in women	X	B
Core indicator	Colorectal cancer incidence	X	F
	Cervical cancer incidence	X	F
Core indicator	Lung cancer incidence	X	F
	**Thematic area 13 – Mental illnesses**		
Development indicator	Mental health problems in children and adolescents	X	B and F
	Sleep- disorders	X	B and F
	Depressive symptoms	X	B and F
Core indicator	Outpatient diagnoses of mental disorders	X	B and F
	Work incapacity due to mental disorders	X	B
	Rehabilitation due to mental disorders	X	B and F
	Early retirement due to mental disorders	X	B and F
	Suicide	X	B
	**Thematic area 14 – Life expectancy and premature mortality**		
Core indicator	Life expectancy and healthy life expectancy	X	B
Core indicator	Premature mortality	X	B

^1^ Core indicators are those in the indicator system that are primarily recommended for completion with underlying data.

^2^ Development indicators are those in the indicator system for which operationalisation has not yet been fully developed and/or suitable data sources have not been identified. **Classification:** A: Causes of climate change B: Health impacts of climate change C: Climate mitigation measures D: Climate adaptation measures E: Particularly vulnerable groups due to socioeconomic factors F: Particularly vulnerable groups due to health-related factors G: Ethical aspects

## References

[1] Hertig E Hunger I Kaspar-Ott I Matzarakis A Niemann H Schulte-Droesch L. Climate change and public health in Germany – An introduction to the German status report on climate change and health 2023. J Health Monit. 2023;8(S3):6–32. doi: 10.25646/11400. 10.25646/11400 PMC10278374 37342432

[2] Sachverständigenrat zur Begutachtung der Entwicklung im Gesundheitswesen. Resilienz im Gesundheitswesen. Wege zur Bewältigung künftiger Krisen. Gutachten 2023. Berlin: Medizinisch Wissenschaftliche Verlagsgesellschaft 2023. p. 25–57.

[3] Winklmayr C Muthers S Niemann H Mücke HG an der Heiden M. Heat-related mortality in Germany from 1992 to 2021. Dtsch Arztebl Int. 2022(119):451–7. doi: 10.3238/arztebl.m2022.0202. 35583101 10.3238/arztebl.m2022.0202 PMC9639227

[4] Romanello M Walawender M Hsu SC Moskeland A Palmeiro-Silva Y Scamman D. The 2024 report of the Lancet Countdown on health and climate change: facing record-breaking threats from delayed action. Lancet. 2024;404(10465):1847–96. doi: 10.1016/S0140-6736(24)01822-1. 39488222 10.1016/S0140-6736(24)01822-1 PMC7616816

[5] Bergmann KC Brehler R Endler C Höflich C Kespohl S Plaza M. Impact of climate change on allergic diseases in Germany. J Health Monit. 2023;8(S4):76–102. doi: 10.25646/11654. 37799537 10.25646/11654 PMC10548488

[6] Breitner-Busch S Mücke HG Schneider A Hertig E. Impact of climate change on non-communicable diseases due to increased ambient air pollution. J Health Monit. 2023;8(S4):103–21. doi: 10.25646/11655.2. 37799533 10.25646/11655.2 PMC10548484

[7] Butsch C Beckers LM Nilson E Frassl M Brennholt N Kwiatkowski R. Health impacts of extreme weather events – Cascading risks in a changing climate. J Health Monit. 2023;8(S4):33–56. doi: 10.25646/11652. 10.25646/11652 PMC10548486 37799532

[8] Günster C Schmuker C. Gesundheit und Klimawandel – welche Potenziale haben versorgungsnahe Daten? Bundesgesundheitsbl. 2024;67(2):155–63. doi: 10.1007/s00103-023-03828-8. 10.1007/s00103-023-03828-8 PMC10834614 38240844

[9] Müller B Haase M Kreienbrink A Schmid S. Klimamigration – Definitionen, Ausmaß und politische Instrumente in der Diskussion. Working Paper 45 der Forschungsgruppe des Bundesamtes. Nürnberg: Bundesamt für Migration und Flüchtlinge; 2012.

[10] Sachverständigenrat für Integration und Migration (SVR), editor. Jahresgutachten 2023. Klimawandel und Migration: was wir über den Zusammenhang wissen und welche Handlungsoptionen es gibt. Berlin: SVR; 2023.

[11] Umweltbundesamt editor. Klimawirkungs- und Risikoanalyse 2021 für Deutschland. Teilbericht 5: Risiken und Anpassung in den Clustern Wirtschaft und Gesundheit. Dessau-Roßlau 2021.

[12] Gesundheitsministerkonferenz. Beschlüsse der GMK 22.06.2022 – 23.06.2022. TOP: 20.2 Befassung der Arbeitsgruppen der AOLG mit dem Thema Klimawandel. 2022 [cited 19.02.2025]. Available from: https://www.gmkonline.de/Beschluesse.html?id=1300&jahr=2022.

[13] Die Träger der Nationalen Präventionskonferenz (NPK). Zweiter Präventionsbericht nach § 20d Abs. 4 SGB V. NPK; 2023 [cited 05.06.2025]. Available from: https://www.npk-info.de/fileadmin/user_upload/ueber_die_npk/downloads/2_praeventionsbericht/zweiter_npk_praeventionsbericht_barrierefrei.pdf.

[14] Unterarbeitsgruppe Präventionsindikatoren der Arbeitsgruppe Gesundheitsberichterstattung, Prävention Rehabilitation und Sozialmedizin (AG GPRS) der Arbeitsgemeinschaft der Obersten Landesgesundheitsbehörden (AOLG). Entwicklung eines Indikatorensystems für die Präventionsberichterstattung der Länder. Diskussionspapier der Unterarbeitsgruppe Präventionsindikatoren. Berlin 2021 [cited 19.02.2025]. Available from: https://www.berlin.de/sen/gesundheit/_assets/gesundheitsberichterstattung/veroeffentlichungen/diskussionspapier_praeventionsindikatoren_runde1.pdf.

[15] The Lancet Countdown. Lancet Countdown: Tracking Progress on Health and Climate Change 2025 [cited 14.04.2025]. Available from: https://lancetcountdown.org/. 10.1016/S0140-6736(16)32124-9 27856085

[16] European Climate and Health Observatory. Indicators on climate change and health. 2024 [cited 11.04.2025]. Available from: https://climate-adapt.eea.europa.eu/en/observatory/evidence/indicators.

[17] Brugger K Delcour J. Integrierte Gesundheitsberichterstattung zu Klima und Gesundheit: Grundlagen für ein Indikatorenset. Grundlagenbericht. Wien: Gesundheit Österreich; 2024 [cited 02.6.2025]. Available from: https://jasmin.goeg.at/id/eprint/3446/1/Integrierte%20GBE%20zu%20Klima%20und%20Gesundheit_bf.pdf.

[18] Australian Institute of Health and Welfare (AIHW). Climate change and environmental health indicators: reporting framework. Canberra AIHW; 2024 [cited 02.06.2025]. Available from: https://www.aihw.gov.au/getmedia/d249dc5d-7c5f-47ec-9b78-da765d00a2fe/aihw-phe-343-climate-change-and-environmental-health-indicators.pdf?v=20240515111427&inline=true.

[19] Robert Koch Institute. MOCCHA (Monitoring of Climate Change-related Health Aspects) – Entwicklung eines Indikatorensets zum Monitoring klimarelevanter Gesundheitsaspekte. 2025 [cited 14.04.2025]. Available from: https://www.rki.de/DE/Themen/Gesundheit-und-Gesellschaft/Klimawandel/Projekte/Klimawandel-MOCCHA-Indikatorenset.html.

[20] Bundesministerium für Umwelt, Naturschutz nukleare Sicherheit und Verbraucherschutz (BMUV). Deutsche Anpassungsstrategie an den Klimawandel 2024. Vorsorge gemeinsam gestalten 2024 [cited 02.06.2025]. Available from: https://www.bmuv.de/DL3369.

[21] Palmeiro-Silva Y Aravena-Contreras R Izcue Gana J González Tapia R Kelman I. Climate-related health impact indicators for public health surveillance in a changing climate: a systematic review and local suitability analysis. The Lancet Regional Health – Americas. 2024;38:100854. doi: 10.1016/j.lana.2024.100854. 39171197 10.1016/j.lana.2024.100854 PMC11334688

[22] World Health Organization (WHO). Fact Sheet: Climate change. 2023 [cited 07.02.2025]. Available from: https://www.who.int/news-room/fact-sheets/detail/climate-change-and-health.

[23] Beermann S Dobler G Faber M Frank C Habedank B Hagedorn P. Impact of climate change on vector- and rodent-borne infectious diseases. J Health Monit. 2023;8(S3):33–61. doi: 10.25646/11401. 10.25646/11401 PMC10278376 37342429

[24] Dupke S Buchholz U Fastner J Förster C Frank C Lewin A. Impact of climate change on waterborne infections and intoxications. J Health Monit. 2023;8(S3):62–77. doi: 10.25646/11402. 37342430 10.25646/11402 PMC10278370

[25] Dietrich J Hammerl JA Johne A Kappenstein O Loeffler C Nöckler K. Impact of climate change on foodborne infections and intoxications. J Health Monit. 2023;8(S3):78–92. doi: 10.25646/11403. 10.25646/11403 PMC10278375 37342431

[26] Meinen A Tomczyk S Wiegand FN Abu Sin M Eckmanns T Haller S. Antimicrobial resistance in Germany and Europe – A systematic review on the increasing threat accelerated by climate change. J Health Monit. 2023;8(S3):93–108. doi: 10.25646/11404. 37342428 10.25646/11404 PMC10278373

[27] Winklmayr C Matthies-Wiesler F Muthers S Buchien S Kuch B an der Heiden M. Heat in Germany: Health risks and preventive measures. J Health Monit. 2023;8(S4):3–32. doi: 10.25646/11651. 10.25646/11651 PMC10548487 37799534

[28] Baldermann C Laschewski G Grooß JU. Impact of climate change on non-communicable diseases caused by altered UV radiation. J Health Monit. 2023;8(S4):57–75. doi: 10.25646/11653. 10.25646/11653 PMC10548485 37799535

[29] Gebhardt N van Bronswijk K Bunz M Müller T Niessen P Nikendei C. Scoping review of climate change and mental health in Germany – Direct and indirect impacts, vulnerable groups, resilience factors. J Health Monit. 2023;8(S4):122–49. doi: 10.25646/11656. 37799536 10.25646/11656 PMC10548489

[30] Bolte G Dandolo L Gepp S Hornberg C Lumbi SL. Climate change and health equity: A public health perspective on climate justice. J Health Monit. 2023;8(S6):3–35. doi: 10.25646/11772. 10.25646/11772 PMC10722520 38105794

[31] Lehrer L Hellmann L Temme H Otten L Hübenthal J Geiger M. Communicating climate change and health to specific target groups. J Health Monit. 2023;8(S6):36–56. doi: 10.25646/11773. 38105792 10.25646/11773 PMC10722519

[32] Mlinarić M Moebus S Betsch C Hertig E Schröder J Loss J. Climate change and public health in Germany – A synthesis of options for action from the German status report on climate change and health 2023. J Health Monit. 2023;8(S6):57–85. doi: 10.25646/11774. 10.25646/11774 PMC10722518 38105793

[33] Walinski A Sander J Gerlinger G Clemens V Meyer-Lindenberg A Heinz A. Auswirkungen des Klimawandels auf die psychische Gesundheit. Dtsch Arztebl Int. 2023;120(8):117–24. doi: 10.3238/arztebl.m2022.0403. 36647584 10.3238/arztebl.m2022.0403 PMC10154789

[34] Haines A. Health co-benefits of climate action. The Lancet Planetary Health. 2017;1(1):e4–e5. doi: 10.1016/S2542-5196(17)30003-7. 29851591 10.1016/S2542-5196(17)30003-7

[35] Wabnitz K Endea M von der Haar A. Evidenzsynthese zu Co-Benefits: Eine Aufarbeitung der aktuellen wissenschaftlichen Evidenz. Berlin: Centre for Planetary Health Policy; 2024 [cited 02.06.2025]. Available from: https://cphp-berlin.de/wp-content/uploads/2024/10/CPHP_Evidenzsynthese_01-2024.pdf.

[36] Umweltbundesamt. Indikatoren und Berichterstattung zur Deutschen Anpassungsstrategie an den Klimawandel (DAS). Hintergrundpapier zum Indikatorenset des Handlungsfelds „Menschliche Gesundheit“. 2023 [cited 10.02.2025]. Available from: https://www.umweltbundesamt.de/sites/default/files/medien/5612/dokumente/hintergrund-papier_01_ge.pdf.

[37] Pörtner HO Roberts DC Tignor MMB Poloczanska E Mintenbeck K Alegría A, editors. Climate Change 2022: Impacts, Adaptation and Vulnerability. Contribution of Working Group II to the Sixth Assessment Report of the Intergovernmental Panel on Climate Change Cambridge, UK and New York, USA: Cambridge University Press 2022.

[38] Zhang X Brandt M Tong X Ciais P Yue Y Xiao X. A large but transient carbon sink from urbanization and rural depopulation in China. Nature Sustainability. 2022;5(4):321–8. doi: 10.1038/s41893021-00843-y.

[39] Maheshwari B Pinto U Akbar S Fahey P. Is urbanisation also the culprit of climate change? – Evidence from Australian cities. Urban Climate. 2020;31:100581. doi: 10.1016/j.uclim.2020.100581.

[40] Nazish A Abbas K Sattar E. Health impact of urban green spaces: a systematic review of heat-related morbidity and mortality. BMJ Open. 2024;14(9):e081632. Epub 20241022. doi: 10.1136/bmjopen-2023081632. 10.1136/bmjopen-2023-081632 PMC11499757 39438088

[41] Ahrens GA Becker U Böhmer T Richter F Wittwer R. Potenziale des Radverkehrs für den Klimaschutz. Dessau/Roßlau: Umweltbundesamt; 2012 [cited 02.06.2025]. Available from: https://www.umweltbundesamt.de/sites/default/files/medien/461/publikationen/4451.pdf.

[42] Canzler W. Voraussetzung für einen wirksamen Klimaschutz: Die Verkehrswende in den Städten. In: Lozán JL Breckle SW Graßl H Kuttler W Matzarakis A, editors. Warnsignal Klima: Die Städte. Hamburg: Verlag Wissenschaftliche Auswertungen in Kooperation mit GEO Magazin-Hamburg; 2019. p. 286–92.

[43] Sterner J Gansefort D Jakobs N. Praktische Herausforderungen und Perspektiven integrierter Strategien für Gesundheitsförderung, Klimaschutz und -anpassung in Kommunen. In: Hartung S Wihofszky P, editors. Gesundheit und Nachhaltigkeit. Berlin, Heidelberg: Springer; 2024. p. 1–9.

[44] Andresen EC Hjelkrem AGR Bakken AK Andersen LF. Environmental Impact of Feeding with Infant Formula in Comparison with Breastfeeding. Int J Environ Res Public Health. 2022;19(11). Epub 20220524. doi: 10.3390/ijerph19116397. 10.3390/ijerph19116397 PMC9180168 35681983

[45] Karlsson JO Garnett T Rollins NC Röös E. The carbon footprint of breastmilk substitutes in comparison with breastfeeding. J Clean Prod. 2019;222:436-45. doi: 10.1016/j.jclepro.2019.03.043. 31190697 10.1016/j.jclepro.2019.03.043 PMC6472111

[46] Pérez-Escamilla R Moran VH. Maternal and child nutrition must be at the heart of the climate change agendas. Matern Child Nutr. 2023;19(1):e13444. Epub 20221019. doi: 10.1111/mcn.13444. 36259528 10.1111/mcn.13444 PMC9749600

[47] World Health Organization (WHO). Tobacco and its environmental impact: an overview. Geneva: WHO; 2017 [cited 02.06.2025]. Available from: https://iris.who.int/bitstream/handle/10665/255574/9789241512497-eng.pdf.

[48] Roy A. A panel data study on the role of clean energy in promoting life expectancy. Dialogues in Health. 2025;6:100201. doi: 10.1016/j.dialog.2024.100201. 39830724 10.1016/j.dialog.2024.100201 PMC11741050

[49] Espinel Z Shultz JM Aubry VP Abraham OM Fan Q Crane TE. Protecting vulnerable patient populations from climate hazards: the role of the nation‘s cancer centers. J Natl Cancer Inst. 2023;115(11): 1252–61. doi: 10.1093/jnci/djad139. 37490548 10.1093/jnci/djad139 PMC11009498

[50] Bell SA Banerjee M Griggs JJ Iwashyna TJ Davis MA. The Effect of Exposure to Disaster on Cancer Survival. J Gen Intern Med. 2020; 35(1):380–2. doi: 10.1007/s11606-019-05465-x. 31659658 10.1007/s11606-019-05465-x PMC6957642

[51] Khan A Plana-Ripoll O Antonsen S Brandt J Geels C Landecker H. Environmental pollution is associated with increased risk of psychiatric disorders in the US and Denmark. PLoS Biol. 2019;17(8): e3000353. Epub 20190820. doi: 10.1371/journal.pbio.3000353. 31430271 10.1371/journal.pbio.3000353 PMC6701746

[52] Braithwaite I Zhang S Kirkbride JB Osborn DPJ Hayes JF. Air Pollution (Particulate Matter) Exposure and Associations with Depression, Anxiety, Bipolar, Psychosis and Suicide Risk: A Systematic Review and Meta-Analysis. Environ Health Perspect. 2019;127(12):126002. Epub 20191218. doi: 10.1289/ehp4595. 31850801 10.1289/EHP4595 PMC6957283

[53] Mambrey V Wermuth I Böse-O‘Reilly S. Extreme weather events and their impact on the mental health of children and adolescents. Bundesgesundheitsbl. 2019;62(5):599–604. doi: 10.1007/s00103-01902937-7. 10.1007/s00103-019-02937-7 30976819

[54] Clayton S. Climate anxiety: Psychological responses to climate change. J Anxiety Disord. 2020;74:102263. Epub 20200626. doi: 10.1016/j.janxdis.2020.102263. 32623280 10.1016/j.janxdis.2020.102263

[55] Zhou L He C Kim H Honda Y Lee W Hashizume M. The burden of heat-related stroke mortality under climate change scenarios in 22 East Asian cities. Environ Int. 2022;170:107602. Epub 20221025. doi: 10.1016/j.envint.2022.107602. 36323066 10.1016/j.envint.2022.107602

[56] Kuch B. Der Einfluss des Klimawandels auf das Auftreten von HerzKreislauf-Erkrankungen. Handlungsansätze und die besondere Herausforderung durch Arzneimittelwechselwirkungen. In: Günster C Klauber J Robra BP Schmuker C Schneide A, editors. VersorgungsReport Klima und Gesundheit. Berlin: MWV Medizinisch Wissenschaftliche Verlagsgesellschaft; 2021. p. 53–62.

[57] Nurses Climate Challenge Europe. Climate-Smart Infant Feeding: Part 1 – Breastfeeding. Health Care Without Harm (HCWH); 2022 [cited 02.06.2025]. Available from: https://www.breastfeedingnetwork.org.uk/wp-content/uploads/2022/08/2022-05-03-NCCEurope-climate-smart-infantfeeding-part1.pdf.

[58] Goodchild M Nargis N Tursan d‘Espaignet E. Global economic cost of smoking-attributable diseases. Tob Control. 2018;27(1):58–64. Epub 20170130. doi: 10.1136/tobaccocontrol-2016-053305. 28138063 10.1136/tobaccocontrol-2016-053305 PMC5801657

[59] Deutsche Gesellschaft für Ernährung e. V. (DGE). DGE-Qualitätsstandard für die Verpflegung in Kitas. Bonn: DGE; 2023 [cited 02.06.2025]. Available from: https://www.dge.de//fileadmin/dok/gemeinschaftsgastronomie/dge-qualitaetsstandards/2023/230929-DGE-QST-Kita.pdf.

[60] Deutsche Gesellschaft für Ernährung e. V. (DGE). DGE-Qualitätsstandard für die Verpflegung in Schulen. Bonn: DGE; 2023 [cited 02.06.2025]. Available from: https://www.dge.de//fileadmin/dok/gemeinschaftsgastronomie/dge-qualitaetsstandards/2023/230928-DGEQST-Schule.pdf.

[61] Deutsche Gesellschaft für Ernährung e. V. (DGE). DGE-Qualitätsstandard für die Verpflegung in Betrieben, Behörden und Hochschulen. Bonn: DGE; 2023 [cited 02.06.2025]. Available from: https://www.dge.de//fileadmin/dok/gemeinschaftsgastronomie/dge-qualitaetsstandards/2023/231023-DGE-QST-Betriebe-DE.pdf.

[62] Deutsche Gesellschaft für Ernährung e. V. (DGE). DGE-Qualitätsstandard für die Verpflegung mit „Essen auf Rädern“ und in Senioreneinrichtungen. Bonn: DGE; 2023 [cited 02.06.2025]. Available from: https://www.dge.de//fileadmin/dok/gemeinschaftsgastronomie/dge-qualitaetsstandards/2023/231005-DGE-QST-Senioren.pdf.

[63] Meier T Christen O. Environmental impacts of dietary recommendations and dietary styles: Germany as an example. Environ Sci Technol. 2013;47(2):877–88. Epub 20121217. doi: 10.1021/es302152v. 23189920 10.1021/es302152v

[64] Springmann M Clark M Mason-D‘Croz D Wiebe K Bodirsky BL Lassaletta L. Options for keeping the food system within environmental limits. Nature. 2018;562(7728):519–25. Epub 20181010. doi: 10.1038/s41586-018-0594-0. 30305731 10.1038/s41586-018-0594-0

[65] Weltgesundheitsorganisation (WHO), Regionalbüro für Europa. Gesundheitshinweise zur Prävention hitzebedingter Gesundheitsschäden. Neue und aktualisierte Hinweise für unterschiedliche Zielgruppen. Kopenhagen: WHO, Regionalbüro für Europa; 2019 [cited 02.06.2025]. Available from: https://iris.who.int/bitstream/handle/10665/341625/WHO-EURO-2021-2510-42266-58732-ger.pdf?sequence=1&isAllowed=y.

[66] Beyersdorff S Lauer M Petruz S. Die gute gesunde Kita gestalten. Referenzrahmen zur Qualitätsentwicklung im Berliner Landesprogramm für die gute gesunde Kita. Berlin Senatsverwaltung für Bildung, Jugend und Familie; 2019 [cited 02.06.2025]. Available from: https://gute-gesunde-kitas-in-berlin.de/app/uploads/2019/12/SBJF-19-002-Referenzrahmen-gute-gesunde-Kita-RZ-23.11.19.pdf.

[67] Gesundheitsamt Rhein-Sieg-Kreis, Abteilung Koordination der Gesundheitsförderung. Klimagesundheit in Kita und Schule. 2025 [cited 14.04.2025]. Available from: https://www.rhein-sieg-kreis.de/micosites/gesundheitsfoerderung/informationen/klimagesund/Uebersicht-klimagesundheit.php.

[68] Deutscher Paritätischer Wohlfahrtsverband – Gesamtverband e. V. Klimaschutz und Klimaanpassung in Kindertageseinrichtungen. Berlin 2022 [cited 02.06.2025]. Available from: https://www.der-paritaetische.de/fileadmin/user_upload/221214_Broschuere_Klimaschutz-Anpassung_in_Kitas_Web.pdf.

[69] Herrmann A Eichinger M. Klimawandel und Gesundheitsförderung. Leitbegriffe der Gesundheitsförderung und Prävention. Glossar zu Konzepten, Strategien und Methoden. 2022. doi: 10.17623/BZGA:Q4-i156-1.0.

[70] Pädagogisches Landesinstitut Rheinland-Pfalz. Klima und Gesundheit. 2025 [cited 14.04.2024]. Available from: https://bildung.rlp.de/gesundeschule/gesunde-schule/themenfelder/klima-und-gesundheit.

[71] Deutsche Gesetzliche Unfallversicherung. Schutz vor UV-Strahlung und Hitze 2025 [cited 14.04.2025]. Available from: https://www.sichere-schule.de/sportfreiflaechen/lehrkraft/uv-schutz.

[72] Ständige Impfkommission. Empfehlungen der Ständigen Impfkommission (STIKO) beim Robert Koch Institute 2025. Epid Bull. 2025;4:1–75. doi: 10.25646/12971.4.

[73] Yamaguchi J Kinoshita K. The threat of a new tetanus outbreak due to urban flooding disaster requires vigilance: a narrative review. Acute Med Surg. 2023;10(1):e839. Epub 20230416. doi: 10.1002/ams2.839. 37077453 10.1002/ams2.839 PMC10106935

[74] Frumkin H Hess J Luber G Malilay J McGeehin M. Climate change: the public health response. Am J Public Health. 2008;98(3):435–45. Epub 20080130. doi: 10.2105/ajph.2007.119362. 18235058 10.2105/AJPH.2007.119362 PMC2253589

[75] Lavigne E Gasparrini A Wang X Chen H Yagouti A Fleury MD. Extreme ambient temperatures and cardiorespiratory emergency room visits: assessing risk by comorbid health conditions in a time series study. Environmental Health. 2014;13(1):5. doi: 10.1186/1476-069X-13-5. 24484632 10.1186/1476-069X-13-5 PMC3922624

[76] Zhao Q Guo Y Ye T Gasparrini A Tong S Overcenco A. Global, regional, and national burden of mortality associated with non-optimal ambient temperatures from 2000 to 2019: a three-stage modelling study. Lancet Planet Health. 2021;5(7):e415–e25. doi: 10.1016/s2542-5196(21)00081-4. 34245712 10.1016/S2542-5196(21)00081-4

[77] Münzel T Daiber A Hahad O. Bedeutung der Umwelt – Luftverschmutzung, Lärm und Hitze als kardiovaskuläre Risikofaktoren. Aktuelle Kardiologie. 2023;12(02):113–9. doi: 10.1055/a-1978-6169.

[78] Hassan AM Nogueira L Lin YL Rogers JE Nori-Sarma A Offodile AC 2nd. Impact of Heatwaves on Cancer Care Delivery: Potential Mechanisms, Health Equity Concerns, and Adaptation Strategies. J Clin Oncol. 2023;41(17):3104–9. doi: 10.1200/jco.22.01951. 37098249 10.1200/JCO.22.01951

[79] Cianconi P Betrò S Janiri L. The Impact of Climate Change on Mental Health: A Systematic Descriptive Review. Front Psychiatry. 2020;11:74. Epub 20200306. doi: 10.3389/fpsyt.2020.00074. 32210846 10.3389/fpsyt.2020.00074 PMC7068211

[80] Hwong AR Wang M Khan H Chagwedera DN Grzenda A Doty B. Climate change and mental health research methods, gaps, and priorities: a scoping review. Lancet Planet Health. 2022;6(3):e281–e91. doi: 10.1016/s2542-5196(22)00012-2. 35278392 10.1016/S2542-5196(22)00012-2

[81] Heinz A Meyer-Lindenberg A Heinz A Meyer-Lindenberg A Adli M Bornheimer B. Klimawandel und psychische Gesundheit. Positionspapier einer Task-Force der DGPPN. Der Nervenarzt. 2023;94(3):225–33. doi: 10.1007/s00115-023-01457-9. 36820855 10.1007/s00115-023-01457-9 PMC9992044

[82] Poore J Nemecek T. Reducing food‘s environmental impacts through producers and consumers. Science. 2018;360(6392):987–92. doi: 10.1126/science.aaq0216. 29853680 10.1126/science.aaq0216

[83] Searchinger TD Wirsenius S Beringer T Dumas P. Assessing the efficiency of changes in land use for mitigating climate change. Nature. 2018;564(7735):249–53. Epub 20181212. doi: 10.1038/s41586-018-0757-z. 30542169 10.1038/s41586-018-0757-z

[84] Clark MA Domingo NGG Colgan K Thakrar SK Tilman D Lynch J. Global food system emissions could preclude achieving the 1.5° and 2°C climate change targets. Science. 2020;370(6517):705–8. doi: 10.1126/science.aba7357. 33154139 10.1126/science.aba7357

